# Evaluation of the effect of photobiomodulation on joint range of motion in dogs

**DOI:** 10.1007/s10103-025-04553-1

**Published:** 2025-06-24

**Authors:** Daniela Duarte, J. C. Alves

**Affiliations:** 1 Faculty of Veterinary Medicine, Lusófona University, Lisbon, Portugal; 2 Divisão de Medicina Veterinária, Guarda Nacional Republicana (GNR), Rua Presidente Arriaga, 9, 1200-771 Lisbon, Portugal; 3 Centro de Ciência Animal e Veterinária, Lusófona University, 1749-024 Lisbon, Portugal; 4 I-MVET – Faculty of Veterinary Medicine, Lusófona University, Lisbon University Centre, Lisbon, Portugal; 5 MED – Mediterranean Institute for Agriculture, Environment and Development, Instituto de Investigação e Formação Avançada, Universidade de Évora, Pólo da Mitra, Ap. 94, 7006-554 Évora, Portugal

**Keywords:** Photobiomodulation therapy, Dog, Joint, Joint range of motion, Digital thermography

## Abstract

To evaluate the effect of photobiomodulation therapy on healthy joint’s range of motion (ROM). Sixteen police working dogs were selected. The limbs of one side of the body were randomly assigned to the treatment group, while the limbs of the other side formed the control group. Elbow, stifle, and tarsal flexion and extension were evaluated, and measurements were made in triplicate by two evaluators, one experienced and one novice. After the initial evaluation, the treated side’s joints underwent PBMT. The dogs had a 5-minute rest before the joint ROM was again measured. Digital thermography was also used to assess the joints before and immediately after PBMT, and after the 5-minute rest period. The variability of the joint median measurements was compared using 1-tail t-tests, and the effect size was determined. Following PBMT, significant differences in ROM were observed in all joints (*p* < 0.01) with a large effect size (0.84 to 0.96). Additionally, digital thermography values showed significant differences in all joints after PBMT (*p* < 0.01), with an increase of up to 5ºC, with a small to medium effect size (0.31–0.61). A significant difference was found for the stifle and tarsus after the 5 min (*p* < 0.01). There were no differences in the measurements by the two investigators. PBMT increased ROM and tissue temperature. This suggests that joint mobilization exercises can be improved by and should be conducted after PBMT. There was no significant difference between the measurements of experienced and novice evaluators.

## Introduction

Photobiomodulation therapy (PBMT) is frequently used in animal rehabilitation due to its noninvasive nature and lack of adverse effects [1, 2]. This therapy involves lasers that emit monochromatic, coherent, and collimated electromagnetic radiation, which allows the laser light to penetrate tissues and stimulate non-harmful and non-thermal cell reactions, resulting in positive therapeutic effects, including pain and inflammation alleviation, immunomodulation, and the promotion of wound healing and tissue regeneration [3–5]. PBMT also produces tissue warming and, consequently, thermal effects, which are correlated to the magnitude of tissue warming. An increase in temperature increases collagen tissue extensibility, thus improving the flexibility of the tissues, which has clinical significance when therapeutic rehabilitation exercises and modalities are performed [6].

To maintain optimal movement and daily activities, adequate joint motion is essential. In veterinary rehabilitation, passive ROM exercises are commonly utilized to promote cartilage nutrition, reduce tissue adhesion, decrease swelling, and relieve pain [7, 8]. The ROM is influenced by a combination of factors, including the joint shape, joint capsule, ligaments, and surrounding tendons and muscles. Normal ROM values vary according to the dog’s size, breed, and body type [9, 10]. ROM is closely linked to flexibility and is susceptible to the effects of physical activity, overall physical condition, and joint health. Specific exercises can be designed to target and improve joint flexibility. An example is passive joint manipulation, which caregivers can conduct [10]. This form of manipulation also improves joint movement and has the potential to contribute positively to tissue healing [7]. Goniometry is a simple, affordable, and noninvasive method, to measure joint angles and quantifies range of motion with excellent precision and repeatability [7].

Digital thermography is a non-contact, noninvasive tool for assessing soft tissue injuries in humans and animals based on heat resulting from physiological functions related to skin temperature regulation [11, 12]. This technology works by detecting variations in tissue heat caused by modifications in structure and function. The thermal patterns are displayed on a color map, with the warmest regions shown in white or red and the cooler regions in blue or black [13]. It can be used as diagnostic screening, enhancing interpretation of physical examinations, guiding therapeutic management, and assessing long-term response to treatment [14]. In human and equine medicine, thermography has proven beneficial, particularly as it does not require anesthesia and does not expose the patient to radiation [13]. It has been extensively reported in canine medicine, from the initial and follow-up evaluation of osteoarthritis patients, including different joints, to tendinopaties [15, 16].

This study aimed to measure the changes in joint range of motion immediately after therapeutic PBMT and once the body returns to its normal temperature. We hypothesized that PBMT could increase joint range of motion and tissue temperature, as measured by digital thermography. Additionally, we aimed to determine if there is any significant difference between the measurements taken by experienced and novice evaluators.

## Materials and methods

The study protocol was approved by the ethical review committee of the Faculty of Veterinary Medicine, Lusófona University (Comissão de Ética e Bem Estar Animal - CEBEA, approval nº 07/2024) and complied with the relevant institutional and national guidelines for the care and use of animals. The ARRIVE guidelines for reporting were followed. Written, informed consent was obtained from the Institution responsible for the animals.

A sample size of 16 dogs was determined before the onset of the study by conducting a statistical power analysis (type-1 error, 0.05; type-2 error, 0.8) to determine the minimal number of dogs necessary to perform statistical comparisons between study groups. Animals were selected from the population of police working dogs of the Guarda Nacional Republicana. Written and informed consent was obtained from the Institution. The inclusion criteria were that dogs were ≥ 18 months of age, with no direct blood relationship with other dogs in the study, no lameness or history of orthopedic disease or trauma, normal orthopedic examination, and no radiographic evidence of joint disease in the considered joints.

The thoracic and pelvic limbs from one side were used as the treatment group (PBMTG), and the contralateral limbs comprised the control group (CG). The side allocated to each group was randomly determined using statistical analysis software. The joint range of motion over the sagittal plain (in flexion and extension) for the elbow, stifle, and tarsal joints were evaluated. Two experienced and novice evaluators independently made goniometric measurements of each dog awake. Investigators were blinded to treatment and control groups. The evaluation of joint ROM has been described before [17]. Briefly, the arms of a transparent plastic goniometer were aligned with anatomic landmarks on the limbs. Before joint angle measurement, each joint was moved through a complete range of motion to determine the joint rotation axis. The center of the goniometer was placed over that axis. All measurements were made in triplicate and recorded. Joints were evaluated one at a time in the order outlined above. After the first evaluation, the joint from the treated side was submitted to PBMT. PBMT parameters are summarized in Table 1. An independent investigator performed PBMT treatment. After PBMT, dogs were allowed a 5-minute rest period, during which they were not manipulated. After the 5 min, joint ROM was again measured in both the treated and control joints.

**Table 1:** Photobiomodulation therapy treatment parameters

	Light Parameters (Dose)		
	Elbow	Stifle	Tarsus
Wavelength (nm)	980 nm	980 nm	980 nm
Radiant Power (W)	10.5	12	8
Irradiance (W/cm2) at skin surface	2.1	2.4	1.6
Fluence (J/cm^2^)	15	15	15
Total Joules	3750	5250	1875
Treatment Protocol	Continuously moving grid pattern ON contact at a speed of 1–3 inches/second according to manufacturer recommendations	Continuously moving grid pattern ON contact at a speed of 2,5–7,5 cm/s according to manufacturer recommendations	Continuously moving grid pattern ON contact at a speed of 2,5–7,5 cm/s according to manufacturer recommendations
Treatment Area (cm^2^)	250 (entire elbow area)	350 (entire stifle area)	125 (entire tarsus area)
Treatment Time	5 min, 57 s	7 min, 18 s	3 min, 54 s

For the collection of the digital thermography images, the same procedure as described before was used [18–20]. The animals were positioned standing in an upright position, as symmetrically as possible. The region of interest was not touched. No fur clipping was performed before image collection. Three images were collected from each joint: before PBMT, immediately after PBMT, and after the 5-minute period. Image settings were adjusted to include a range of 15–40 °C and an emissivity of 0.98. An HT-19 Thermal Imager camera (HTI) was used, and thermographic images were analyzed with IR Image Tools (HTI). A Rainbow HC color pallet was selected, and equal-sized temperature boxes were placed on the joint’s anatomical area. Mean and maximal temperatures were determined. Only mild restraint was required to perform all the procedures.

To evaluate intertester variability, the median measurements for the 2 investigators were compared using paired t-tests for joint extension and flexion. To assess goniometric measurements of each joint, the variability of mean measurements in flexion and extension between groups was compared with a 1-tail t-test. The variability of mean measurements of digital thermography between groups tarsus, stifle, and elbow joints was compared using 1-tail t-tests. The effect size was determined and considered to be small if *r* < 0.3, medium if 0.3 < *r* < 0.5, and large if *r* > 0.5. Results were analyzed with IBM SPSS Statistics version 20, *p* < 0.05.

## Results

The sample included 16 dogs, with a mean body weight of 25.3 ± 2.6 kg and a mean age of 10.6 ± 3.4 years. Both sexes were represented, with 9 males and 6 females, and four breeds were represented: Belgian Malinois Shepherd Dogs (*n* = 6), Labrador Retriever (*n* = 5), Dutch Shepherd Dogs (*n* = 3), and German Shepherd Dogs (*n* = 2).

There were no significant differences between the two groups at the initial evaluation. The results of measurements made by the 2 investigators did not differ significantly (ranging from 0.20 to 0.39). For that reason, the results presented are the result of the measurements performed by both investigators. Table 2 presents values for joint ROM in healthy elbows (before PBMT was performed), stifle, and tarsus. Table 3 presents values for joint ROM in CG and PBMTG. A significant difference was observed in all joints after PBMT, with a medium to large effect size.

**Table 2:** Joint range of motion (mean and standard deviation) of normal elbows, stifles, and Tarsus (n indicates individual joints, i.e., each dog contributes with two joints each)

	Elbow				Stifle				Tarsus			
	Flexion		Extension		Flexion		Extension		Flexion		Extension	
	Mean	SD	Mean	SD	Mean	SD	Mean	SD	Mean	SD	Mean	SD
Overall (*n* = 32)	33.4	6.0	159.6	7.2	41.1	3.6	160.2	9.4	35.8	6.6	158.6	11.8
Belgian Malinois (*n* = 12)	30.0	7.5	162.5	4.6	39.9	3.0	161.2	6.8	32.8	9.7	152.5	16.3
Labrador Retriever (*n* = 10)	35.5	3.7	157.8	6.7	41.9	3.3	159.6	13.3	38.2	1.6	163.5	5.2
Dutch Shepherd (*n* = 6)	37.6	2.6	155.1	10.2	43.0	2.7	158.7	4.0	35.9	2.7	161.8	2.2
German Shepherd (*n* = 4)	32.4	2.6	162.2	2.4	40.3	0.5	161.0	1.5	39.0	1.2	160.0	5.1

**Table 3:** Joint range of motion (mean, standard deviation, and variation - Δ°) of elbows, stifles, and Tarsus in the control (CG) and treatment (PBMTG) groups before and after photobiomodulation therapy. * indicates significance

Group	Elbow															
	Flexion								Extesion							
	Before PBMT		p	After PBMT		Δ̊	p	ES	Before PBMT		p	After PBMT		Δ̊	p	ES
	Mean	SD		Mean	SD				Mean	SD		Mean	SD			
CG	32.7	6.9	0.53	35.2	3.8	2.5	< 0.01*	0.43	160.6	6.2	0.58	160.2	6.8	-0.4	< 0.01*	0.86
PBMTG	34.2	5.2		30.6	8.1	-3.6			158.6	8.2		167.8	7.2	9.2		
	**Stifle**															
CG	41.4	3.1	0.19	39.2	6.2	-2.2	< 0.01*	0.51	159.1	34.3	0.26	158.7	6.0	-0.4	< 0.01*	0.96
PBMTG	40.9	4.2		34.3	6.6	-6.6			161.3	11.1		166.3	7.1	5.0		
	**Stifle**															
CG	36.9	5.0	0.47	35.7	6.4	-1.2	< 0.01*	0.66	156.5	12.4	0.32	155.5	13.6	-1.0	< 0.01*	0.84
PBMTG	34.8	8.2		31.7	6.6	-3.1			160.8	11.1		165.8	11.1	5.0		

**Fig. 1:**
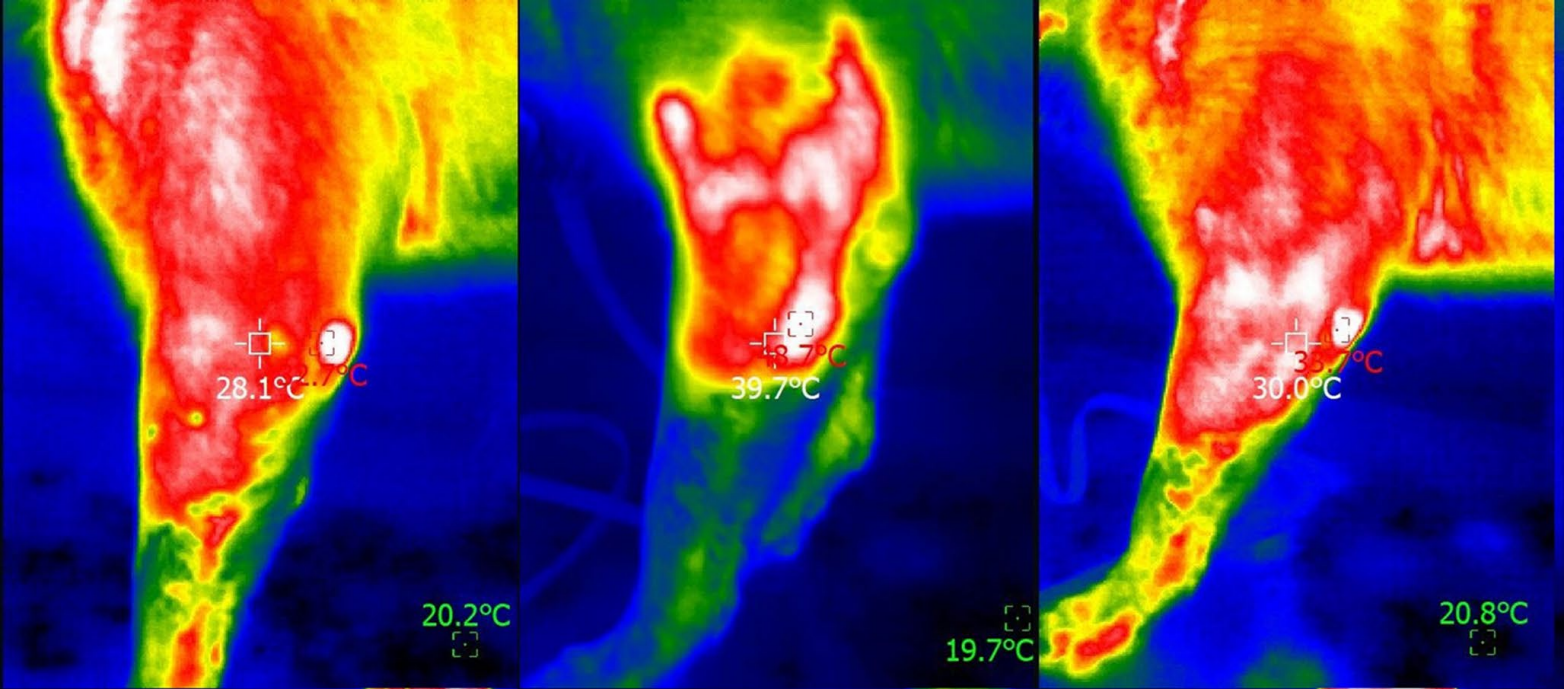
Lateral thermogram of an elbow before (left), immediately after photobiomodulation therapy (center), and after 5 min (right). The head of the animal is to the left of the image

**Fig. 2:**
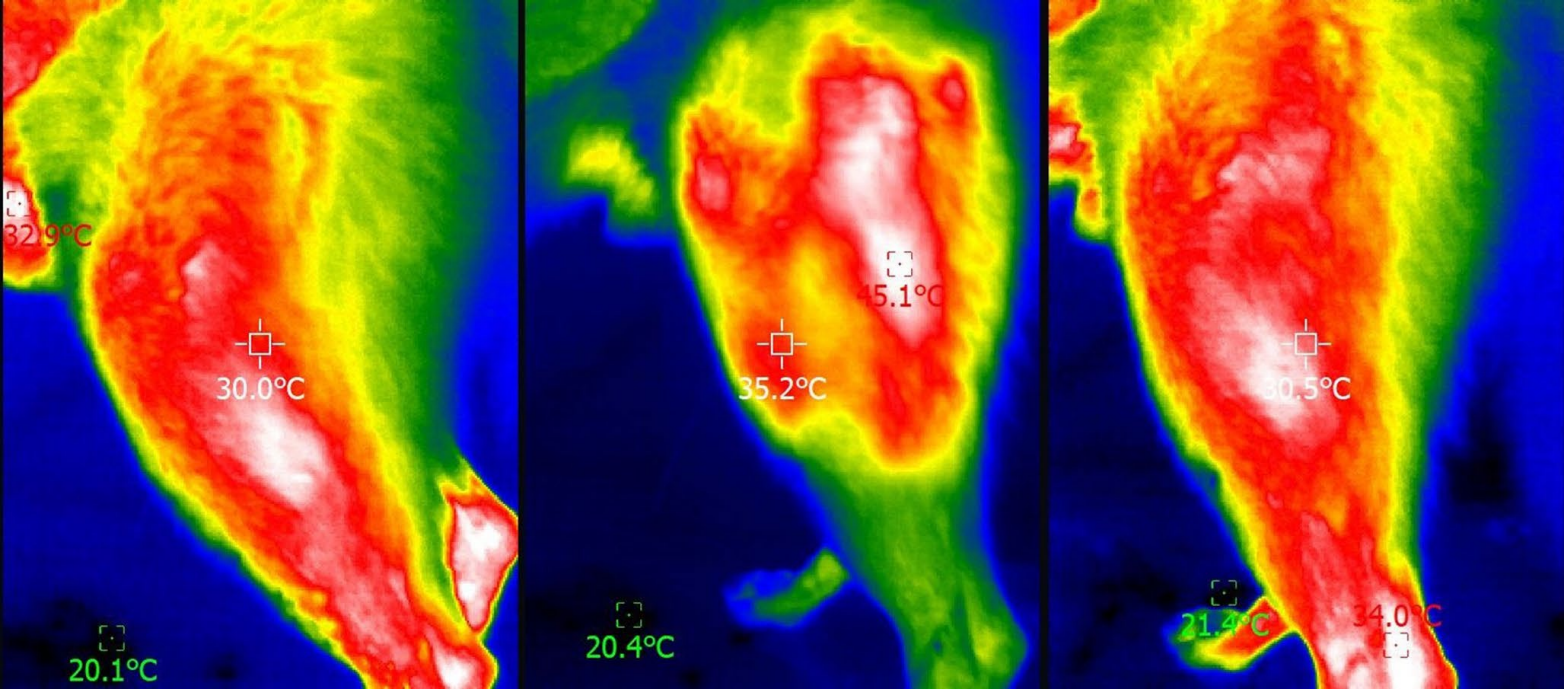
Lateral thermogram of a stifle before (left), immediately after photobiomodulation therapy (center), and after 5 min (right). The head of the animal is to the left of the image

**Fig. 3:**
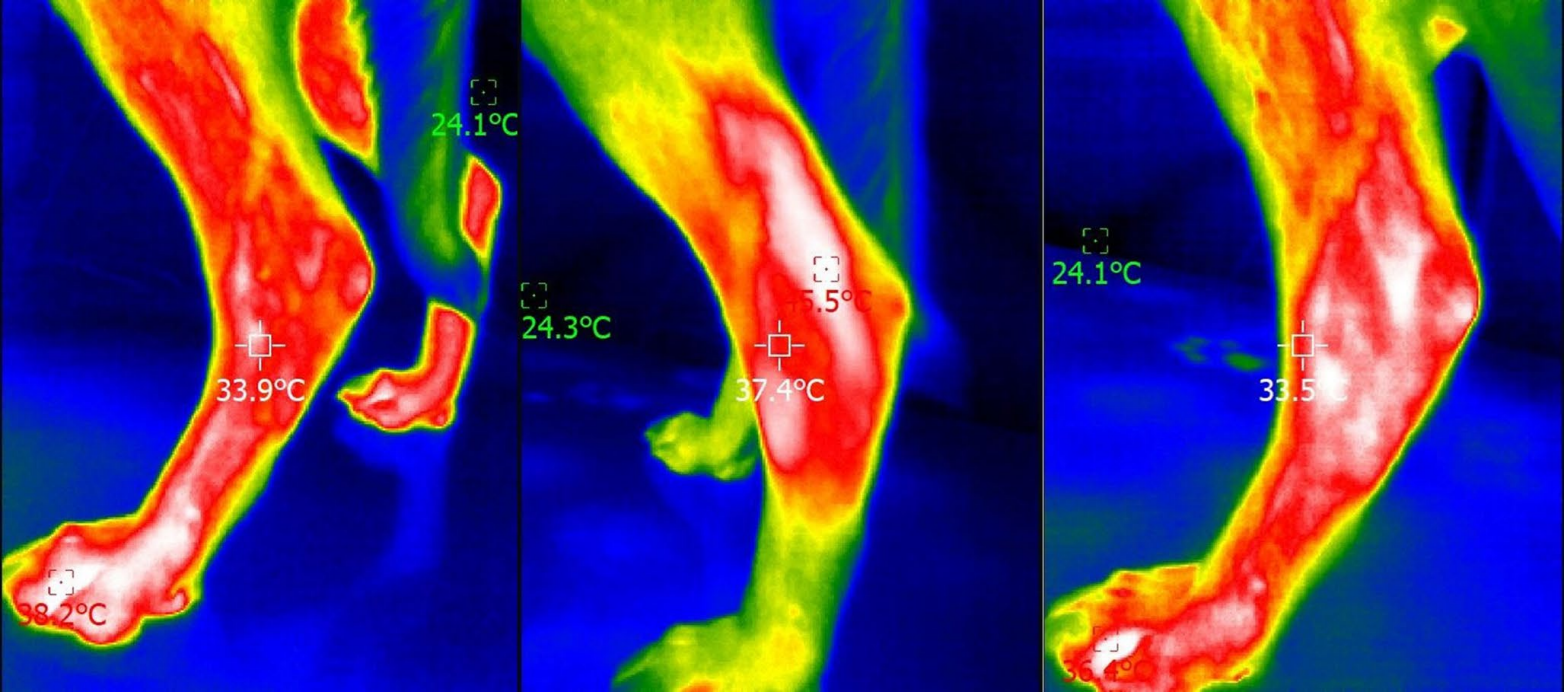
Lateral thermogram of a tarsus before (left), immediately after photobiomodulation therapy (center), and after 5 min (right). The head of the animal is to the left of the image

Digital thermography values of elbows, stifles, and tarsus in the two groups, before and after photobiomodulation therapy and after 5 min, are presented in Table 4. A significant difference was observed in all joints after PBMT, with a small to medium effect size. For the stifle and tarsus, a significant difference was still observed after the 5-minute period. Figures 1, 2 and 3 show a lateral thermogram of an elbow, stifle, and tarsus (respectively) before (left), immediately after photobiomodulation therapy (center), and after 5 min (right).

**Table 4:** Digital thermography values (mean, standard deviation, and variation - ΔT) of elbows, stifles, and Tarsus in the control (CG) and treatment (PBMTG) groups before and after photobiomodulation therapy and after 5 min. * indicates significance

Group	Elbow												
	Before PBMT			After PBMT					After 5-minutes				
	Mean	SD	p	Mean	SD	ΔT	p	ES	Mean	SD	ΔT	p	ES
CG	32.9	1.3	0.11	33.1	1.6	0.2	< 0.01*	0.61	32.6	1.5	-0.3	0.45	0.16
PBMTG	33.1	1.8		38.1	3.4	5.0			32.2	4.1	-0.9		
**Group**	**Stifle**												
	**Before PBMT**			**After PBMT**					**After 5-minutes**				
	**Mean**	**SD**	**p**	**Mean**	**SD**	**ΔT**	**p**	**ES**	**Mean**	**SD**	**ΔT**	**p**	**ES**
CG	32.4	1.7	0.26	32.8	2.3	0.4	< 0.01*	0.55	32.1	1.3	-0.3	< 0.01*	0.31
PBMTG	32.6	1.5		36.6	2.9	4.0			34.2	2.1	1.6		
**Group**	**Tarsus**												
	**Before PBMT**			**After PBMT**					**After 5-minutes**				
	**Mean**	**SD**	**p**	**Mean**	**SD**	**ΔT**	**p**	**ES**	**Mean**	**SD**	**ΔT**	**p**	**ES**
CG	33.0	2.3	0.25	32.7	2.1	-0.3	< 0.01*	0.53	32.7	2.3	-0.3	< 0.01*	0.12
PBMTG	32.8	2.6		38.1	3.0	5.3			33.6	1.8	0.8		

## Discussion

Our research shows that PBMT can increase joint ROM of healthy joints and local tissue temperature. While no differences were observed between the two groups at the initial assessment, following PBMT, a significant increase in ROM was observed in all joints (*p* < 0.01) of PBMTG, with a large effect size, ranging from 0.84 to 0.96. A previous report has shown that PBMT can improve joint ROM in dogs with hip OA, but these effects were only observed after 15 to 30 days of treatment, and within session changes were not evaluated [21]. These changes in ROM can be attributed to the effects of PBMT on biological tissues, where light-based therapy stimulates cellular processes. PBMT increases mitochondrial ATP production, reduces oxidative stress, and enhances tissue repair [22]. These mechanisms likely reduce inflammation in the synovial membranes and surrounding soft tissues, such as tendons and ligaments, leading to increased flexibility and decreased stiffness in the joint capsule [23]. This explains the overall improvement in ROM after long-term treatment, in joints where stiffness is often exacerbated by inflammation and structural degeneration, such as cartilage loss and osteophyte formation, which ultimately leads to restricted ROM [23].

However, these are unlikely to be the cause in case for healthy joints and within a session. In disease joints, putting a joint through its ROM can be painfull, and is also limited by sensory factors rather than just mechanical ones [22]. In these cases, pain and inflammation become a central limiting factors, and the mechanical restriction, combined with pain, results in a more limited and variable ROM than healthy joints. The analgesic effect of PBMT can improve patient comfort and improve ROM within the session. Also, the thermal effect resulting from the PBMT session, while not responsible for the beneficial effects of PBMT, has resulted in a “beneficial side-effect”. An increase in temperature increases collagen tissue extensibility, thus improving the flexibility of the tissues, which has clinical significance when therapeutic rehabilitation exercises and modalities are performed [6]. This may be one of the reasons why an increase in ROM was observed in this study.

Previous reports have shown that joints affected by osteoarthritis exhibit increased variability in ROM measurements [24]. There are several reasons for this finding. The pain and inflammation in joints affected by osteoarthritis often lead to reduced movement to avoid discomfort. It also can lead to muscle weakening and joint stiffness over time [25]. Additionally, when evaluating different joints, proximal joints are surrounded by more soft tissue structures, and the larger muscle mass around proximal joints can make it harder to palpate bony landmarks, making adequate goniometric measurements harder [17]. We found no differences between the two evaluators in any considered joints. Even though these were healthy joints, the animals were fully awake and were energetic, which could have been more challenging. To overcome these possible limitations, an adequate preparation was followed beforehand, to make sure that the principles of proper evaluations were understood, particularly given that goniometry is recognized as having a relatively low learning curve [17]. Having a standardized protocol is crucial in minimizing operator variability. By following the established protocols and taking as much time as required to perform the evaluations, and adequately handle the dogs with care and attention to detail, goniometric measurements remained consistent across both researchers. This result is consistent with previously reported [26, 27].

Regarding the results obtained with digital thermography, significant temperature increases were observed in all joints immediately after PBMT (*p* < 0.01), with an increase of up to 5ºC. This rise in temperature can be attributed to the thermal effect of PBMT, which leads to vasodilation, and increased blood flow to the skin and underlying tissues [28]. Raising tissue temperature by more than 3ºC above baseline levels could notably enhance ROM, likely due to collagen’s viscoelastic characteristics [14]. These findings imply that investigating the effects of PBMT on joint temperature could significantly improve patient care [29]. The thermographic findings in the present study align with this observation, suggesting that the increase in temperature contributed to the improved ROM observed following PBMT. This further stresses the broad role of PBMT in rehabilitation. In addition to healing and analgesic effects, which establish PBMT as a therapeutic modality on its own, it can also serve as a preparatory modality for other therapeutic approaches, as joint mobilization or therapeutic exercises.

After the 5 min, the increase in temperature was only observed in the stifle and tarsus (*p* < 0.01). The reason for this is unclear. The temperature rise was similar in the joints considered (4–5ºC). Surrounding muscle masses could have played a role, as the shoulder is surrounded by large muscle masses proximally, but also is the stifle. Future studies should consider measuring muscle girth to evaluate this effect. Coat characteristics could also play a role. A previous study highlighted that German Shepherd dogs exhibited lower values during thermographic evaluation compared to other considered breeds [21]. This factor is balanced by having the same animal serving as a control and treatment. In addition, the remaining breeds included in the sample, particularly Belgian Malinois and Dutch Shepherd, have a similar coat with a thick undercoat.

Despite the recognized advantages, the homogeneity of the sample also has its limitations. The sample was composed exclusively of active police working dogs. These animals have musculoskeletal structures that endure significantly more stress and physical demands than companion dogs. They also exhibit greater muscle masses, joint strength, and overall physical conditioning [27]. Although PBMT parameters were determined based on the patients individual characteristics (as it should always be), future studies should include a more diverse sample of dogs, accounting for variations in size, breed, and physical activity levels. Additionally, future studies should include a longer post-PBMT follow-up to evaluate how long this increase in joint ROM is present.

## Conclusions

Our results show that PBMT can increase joint range of motion in healthy joints. Including PBMT in treatment protocols, especially before joint mobilization exercises, can improve therapeutic outcomes by reducing stiffness and increasing joint flexibility and comfort. No differences were found between goniometric measurements taken by experienced and novice evaluators.

## Data Availability

No datasets were generated or analysed during the current study.
